# Individualization of piperacillin dosage based on therapeutic drug monitoring with or without model-informed precision dosing: a scenario analysis

**DOI:** 10.1093/jac/dkaf007

**Published:** 2025-01-17

**Authors:** David Haefliger, Lynn Mina, Monia Guidi, Catia Marzolini, Paul Thoueille, Laura E Rothuizen, Yann Thoma, Laurent A Decosterd, Benoit Guery, François R Girardin, Thierry Buclin

**Affiliations:** Service of Clinical Pharmacology, Lausanne University Hospital and University of Lausanne, Rue du Bugnon 17, 1011, Lausanne, Switzerland; Centre for Research and Innovation in Clinical Pharmaceutical Sciences, Lausanne University Hospital and University of Lausanne, Lausanne, Switzerland; Service of Clinical Pharmacology, Lausanne University Hospital and University of Lausanne, Rue du Bugnon 17, 1011, Lausanne, Switzerland; Centre for Research and Innovation in Clinical Pharmaceutical Sciences, Lausanne University Hospital and University of Lausanne, Lausanne, Switzerland; Service of Clinical Pharmacology, Lausanne University Hospital and University of Lausanne, Rue du Bugnon 17, 1011, Lausanne, Switzerland; Division of Infectious Diseases and Hospital Epidemiology, University Hospital Basel, University of Basel, Basel, Switzerland; Department of Molecular and Clinical Pharmacology, Institute of Translational Medicine, University of Liverpool, Liverpool, UK; Service of Clinical Pharmacology, Lausanne University Hospital and University of Lausanne, Rue du Bugnon 17, 1011, Lausanne, Switzerland; Service of Clinical Pharmacology, Lausanne University Hospital and University of Lausanne, Rue du Bugnon 17, 1011, Lausanne, Switzerland; School of Engineering and Management Vaud, HES-SO University of Applied Sciences and Arts Western Switzerland, Yverdon-les-Bains, Switzerland; Service of Clinical Pharmacology, Lausanne University Hospital and University of Lausanne, Rue du Bugnon 17, 1011, Lausanne, Switzerland; Service of Infectious Diseases, Lausanne University Hospital and University of Lausanne, Lausanne, Switzerland; Service of Clinical Pharmacology, Lausanne University Hospital and University of Lausanne, Rue du Bugnon 17, 1011, Lausanne, Switzerland; Service of Clinical Pharmacology, Lausanne University Hospital and University of Lausanne, Rue du Bugnon 17, 1011, Lausanne, Switzerland

## Abstract

**Background:**

Model-informed precision dosing (MIPD) combines population pharmacokinetic knowledge with therapeutic drug monitoring (TDM) to optimize dosage adjustment. It could improve target concentration attainment over empirical TDM, still widely practised for broad-spectrum antibiotics.

**Objectives:**

To evaluate the respective performance of TDM and MIPD in achieving target piperacillin exposure.

**Methods:**

Measurements from 80 courses of intermittent piperacillin infusions, each with two TDM samples, were retrospectively submitted to our MIPD software TUCUXI. We considered six dosage adjustment strategies: identical dosage for all (4000 mg q8h), actual initial dosage (chart-based), actual empirical adjustment following first TDM, *a priori* MIPD-based dosage, *a posteriori* MIPD-based adjustment after first TDM and MIPD including both TDM measurements. Dosing strategies were compared regarding daily dosage, trough levels distribution and PTA (with target trough 8–32 mg/L).

**Results:**

Median trough concentration fell within 8–32 mg/L for all strategies except *a priori* MIPD-based dosage (42 mg/L). Distributions of trough concentrations predicted with the six dosage adjustment strategies showed significant differences, with both *a posteriori* MIPD-based strategies best reducing their standard deviation (*P* < 0.001). PTA of 32%, 32%, 55%, 29%, 83% and 94% were estimated, respectively for the six strategies (*P* < 0.001). Poor performance of *a priori* MIPD-based dosage did not hinder *a posteriori* MIPD-based strategies from significantly improving target attainment.

**Conclusions:**

Whilst empirical TDM improves exposure standardization and target attainment compared with no TDM, MIPD can still bring further improvement. Prospective trials remain warranted to confirm MIPD benefits not only on target attainment but also on clinical endpoints.

## Introduction

Optimizing β-lactam antibiotics dosage represents an important pathway towards improving their efficacy and safety, while simultaneously addressing the growing incidence of antimicrobial resistance.^1,2^ The important variability among patients characterizing β-lactam pharmacokinetics (PK) results in a wide range of circulating exposure levels under standard dosage. Low exposure increases the probability of clinical failure and emergence of antimicrobial resistance, while high exposure is associated with an increased likelihood of toxicity.^3,4^ Therapeutic drug monitoring (TDM), consisting of measuring drug concentrations and consequently adjusting the dosage in patients, is advocated as the best approach for maximizing efficacy, minimizing toxicity and preventing antimicrobial resistance. Still, available evidence supporting the clinical usefulness of TDM for β-lactams is scarce.^5,6^ One reason making a clinical benefit difficult to demonstrate lies in the practicalities of interpreting measured concentrations and translating them into dosage adjustment.

Piperacillin, a broad-spectrum β-lactam antibiotic, exhibits time-dependent antimicrobial activity. As for other β-lactams, the PK/PD index of choice that relates exposure with antibacterial effect is the fraction of the dosing interval during which the free plasma drug concentrations remain above the MIC of the bacteria (%fT > MIC).^1^ Exposure thresholds predicting the emergence of antimicrobial resistance or adverse events are less well defined.^3,7–13^ A 50% probability of neurotoxicity has been estimated for trough concentrations above 360 mg/L and of nephrotoxicity above 450 mg/L.^7,8^ Toxicity may occur at even lower concentrations.^12,14^ In many institutions including ours, intermittent short infusions are preferred as they obviate the need for a dedicated venous line. In such regimens, our institutional recommendations aim at maintaining trough levels at steady state above four times the documented MIC if the value is known, otherwise in the range of 8–32 mg/L. Although a crude approximation considering total and not free concentration, a target trough concentration range of 8–32 mg/L ensures 100% T > MIC to 100% T > 4 × MIC for the majority of Enterobacteriaceae identified at our centre (primarily *Escherichia coli* and *Klebsiella pneumoniae*), which have an ECOFF of 8 mg/L according to EUCAST. This range also allows for coverage of most Enterobacteriaceae and *Pseudomonas aeruginosa* strains encountered in our hospital throughout 2021 (Figure S1, available as Supplementary data at *JAC* Online). Nevertheless, if an infection with *P. aeruginosa* is suspected, the target trough concentration is set at 16 mg/L (target range of 16–32 mg/L), following EUCAST.

Population PK (popPK) models predict the average and likely range of exposure expected in a specific patient, considering the patient's characteristics influencing drug disposition.^2^ Model-informed precision dosing (MIPD) software, which combines information from popPK models and individual TDM, is increasingly available.^15,16^ Bayesian estimation allows estimating the *a posteriori* maximum likelihood PK profile of a specific patient based on a single concentration measurement and an established popPK model.^1^ Even in the absence of measurement, only by considering individual variables influential for drug PK, such as age, body weight, and renal function, model-based prescription can support *a priori* individualized patient-tailored antibiotic therapy.^2^ TUCUXI is a novel software application that allows computing both *a priori* and *a posteriori* maximum likelihood individual predictions of drug concentrations based on popPK models, integrating known patient characteristics with observed concentrations.^17,18^ Regarding the popPK of piperacillin, the first step was to identify the model which best applied to our population.

We introduced piperacillin TDM in our institution several years ago. Yet, TDM interpretation and dosage adjustment have been done empirically until presently, without resorting formally to an MIPD strategy. This retrospective study aimed to evaluate on a sample of real-life patients the benefits of TDM without and with MIPD, in terms of standardization of circulating concentration exposure and PTA. Our objectives were to assess our current TDM practice, to compare it with alternative dosage adjustments corresponding to various utilizations of TDM coupled with TUCUXI MIPD software, and to predict the benefits of an MIPD approach based on the most appropriate model for our patients.

## Patients and methods

### Ethics

The utilization of TDM data to characterize drug PK and/or response was approved by the Ethics Commission on human research of the Canton of Vaud (CER-VD ID 2024-01405).

### Identification of a popPK model of piperacillin to implement in TUCUXI

Among several popPK models published for piperacillin, the model developed by Chen *et al*.^19^ was retained and implemented in TUCUXI. Details on the selection, implementation and validation of this popPK model are given in Text and Table S2. In brief, this model was selected among published alternatives identified through a thorough literature search and followed by an external validation step carried out with NONMEM using data from 76 patients who received piperacillin TDM in our centre. The one-compartment model of Chen *et al*.^19^ has population estimates of 13.8 L/h for CL with 31.1% interindividual variability (IIV), 21.7 L for distribution volume (V) with 38% IIV and 9.3% proportional residual error. Creatinine clearance (determined using the Cockcroft–Gault equation) and body weight were identified as covariates affecting piperacillin CL and V, respectively. In our patients, it showed a non-significant mean bias of +6% and an imprecision of 33%. The model was implemented in TUCUXI and concentrations predicted by NONMEM and TUCUXI were further compared: *a priori* and *a posteriori* prediction curves according to both tools were essentially superimposable.

### Study design

For this retrospective observational monocentric study, we collected data from TDM request forms, on which we based our usual TDM interpretation and empirical dosage adjustment advice, without reliance on any MIPD tool. We selected all patients having provided at least two TDM samples, the first used for dosage individualization, and the second for verifying the impact of the performed TDM intervention on piperacillin exposure. Based on both TDM measurements and the popPK model of Chen *et al*.,^19^ we estimated patients’ individual PK parameters with NONMEM. We then simulated various dosage adjustment strategies to evaluate the benefits brought respectively by TDM without and with MIPD: identical dosage for all (4000 mg q8h), actual initial dosage (chart-based), actual empirical adjustment following first TDM, *a priori* MIPD-based dosage, *a posteriori* MIPD-based adjustment after first TDM and MIPD including both TDM measurements.

### Patient population

Among all adult patients (≥18 years) having received a course of piperacillin as intermittent short intravenous infusions at least once between January 2021 and December 2021, we selected those for whom at least two TDM measurements were performed. Patients on renal replacement therapy or receiving piperacillin in continuous infusion were excluded. We recorded prescribed piperacillin dosages, height and body weight, age and sex, renal function and albumin level, microbiological data if available (type of infection, identified bacteria, MIC) and hospital ward. We anonymized data collected during the routine TDM activity before using them for further analyses. TDM samples were drawn in 2.6 mL EDTA-K tubes and transported to the laboratory, usually within 30 minutes. Only total piperacillin plasma concentration was measured by high-performance liquid chromatography with tandem mass spectrometry, using a method developed in-house and fully validated. The lower limit of quantification was 0.08 mg/L and the upper limit 160 mg/L, the accuracy and precision were good.^20^ All reported piperacillin concentrations represent total and not free concentrations. For clarity purposes, the term ‘concentration’ in the following sections will refer specifically to total concentration. Of note, tazobactam, administered alongside piperacillin at a constant dose ratio of 1:8, was not measured (measurement not performed in our centre).

### Intervention

**Figure 1.**
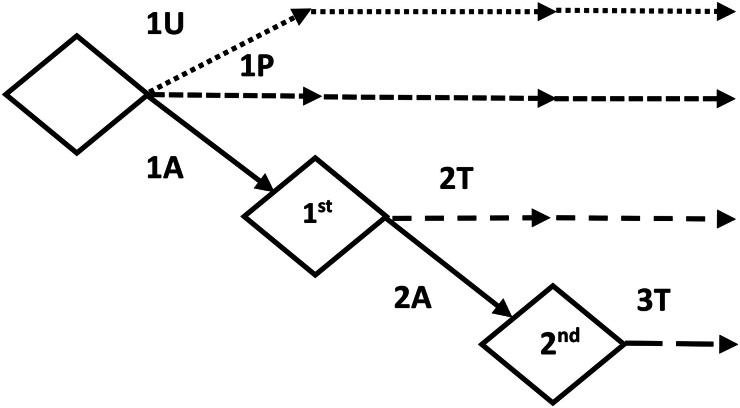
Derivation of the six dosing strategies studied, namely uniform dosage of 4000 mg q8h in all patients (strategy **1U**), actual initial dosage based on a reference dosing chart (strategy **1A**), actual empirical dosage adjustment following first TDM (strategy **2A**), *a priori* MIPD-based dosage (strategy **1P**), *a posteriori* MIPD-based adjustment after first TDM (strategy **2T**) and MIPD-based recommendation based on both TDM measurements (strategy **3T**).

Actual practice was discriminated from simulated alternative dosing strategies (Figure 1). The *actual practice* corresponded to both the initial dosage (chart-based dosage) and the TDM adjustment performed by a clinical pharmacologist not relying on MIPD. We considered two decision steps: (i) actual initial dosage, based on a reference dosing chart (strategy **1A**; Table S3) and (ii) actual dosage adjustment following first TDM (strategy **2A**). The *simulated strategies* were alternatives for the two dosage decision steps, before and after TDM. For the initial dosage, we tested either a uniform dosage of 4000 mg q8h in all patients (strategy **1U**) or an *a priori* MIPD-based dosage obtained after entering the relevant patient’s characteristics into TUCUXI (strategy **1P**). For the dosage adjustment step, we computed an *a posteriori* MIPD-based adjustment with TUCUXI (strategy **2T**). Finally, a third virtual step involving the TUCUXI-based recommendation based on both TDM measurements available (strategy **3T**) was considered as the best adjustment achievable.

### Outcomes

For the six dosage adjustment strategies compared, we estimated various target attainment indices, namely distribution of piperacillin trough levels (median and variance), closeness to the ideal target value of 16 mg/L (allowing *P. aeruginosa* coverage according to EUCAST), attainment of the defined therapeutic range of 8–32 mg/L, and the fraction of the dosage interval covered by concentrations above 8, 16 or 32 mg/L.

### Data analysis

First, we described the collected data (mean, median and percentages), and the outcome indices of interest [observed concentrations and daily doses (DDs)] on both TDM measurements. In addition, we compared the observed concentrations between both TDM cycles. These were available only for actual dosages and not always performed precisely at trough. Second, we compared observed concentrations and model-based predictions from TUCUXI. As such, we compared *a priori* predictions from TUCUXI with the first TDM measurements, and *a posteriori* predictions based on first TDM values with the second TDM measurements by calculating mean log errors (MLEs) and root mean square errors (RMSLEs) on log-transformed values. Third, we described and compared the outcome indices of interest (distribution of through levels, DDs, and dosage interval coverage) resulting from the six dosage adjustment strategies using PK predictions based on the patients’ individual maximum likelihood parameters obtained from their two TDM measurements merged into the model of Chen *et al*.^19^ coded in NONMEM (Text S4). Only model-based extrapolations, available for both actual and simulated dosages, could be compared between all six strategies. We analysed continuous variables with analysis of variance, variance ratio (*F*-test) and Levene's test for equality of variance, Wilcoxon non-parametric tests and Cochran's Q test when applicable, accounting for ‘patient’ effect whenever indicated and setting statistical significance at 2-sided *P* < 0.05. All concentration data were log-transformed for the analyses. Statistical analyses and figures were performed using STATA (v.18, StataCorp 2021) and GraphPad Prism (v. 9.0.0, GraphPad Software, 2020).

## Results

### Study population and observed exposure

**Figure 2.**
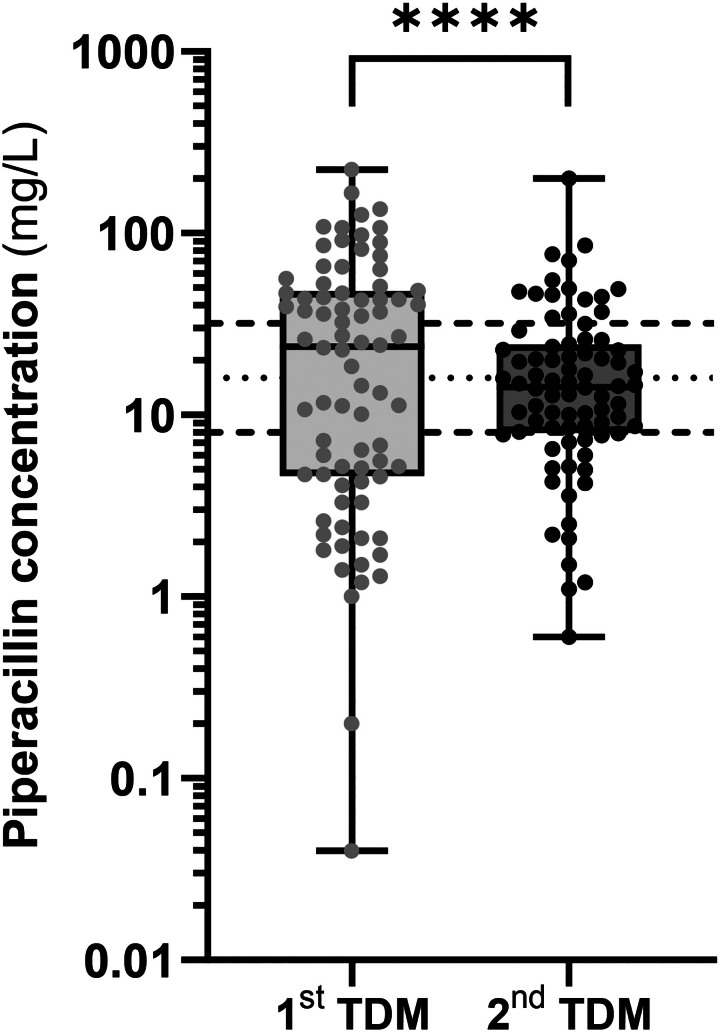
Piperacillin concentrations measured on first and second TDM. The variance ratio (*F* test) on log-transformed values is 64% (****P < 0.001). The single point below the lower limit of quantification (LLOQ = 0.08 mg/L) was treated as LLOQ/2.

As shown in our flow chart (Figure S5), 80 courses with two TDM measurements could be identified in 78 of 261 adults benefiting from piperacillin TDM over the 1-year studied period (71% males; mean ± SD age: 64 ± 16 years; body weight: 79 ± 18 kg; body mass index: 27 ± 7 kg/m^2^; creatinine clearance Cockcroft–Gault: 86 ± 52 mL/min; 59% in ICU). Among the 80 piperacillin treatment courses, albumin levels were documented in 73 courses. The mean ± SD value was 27 ± 4 g/L, with a range from 17 to 43 g/L. Close to 95% of our patients had hypoalbuminemia (albumin level <35 g/L). There were 36% respiratory tract infections, 32% intra-abdominal infections, 17% fevers of unknown origin, 5% skin and soft tissue infections, 5% osteoarticular infections, 4% urinary tract infections and 1% intravascular infections. *Pseudomonas aeruginosa* was identified in 12%. The MIC values were available only in about 14% of our patients with a median value of 4 mg/L (range 1–16 mg/L). Median total piperacillin concentrations were 24.6 mg/L (range: 0.04–224 mg/L) and 14.5 mg/L (range: 0.6–200 mg/L), respectively on the first and second TDM, with a no significant difference. Mean geometric concentrations were 15.2 and 13.4 mg/L, respectively on the first and second TDM. However, as shown in Figure 2, the dispersion of measured concentrations was 36% lower on the second TDM than on the first TDM, with variance of second TDM significantly smaller than that of first TDM (amounting to only 64% of the latter; *P* < 0.001 with variance ratio test). When considering 16 mg/L as the ideal target, measured concentrations were significantly closer to this target on second TDM than on first TDM (closeness of measured concentrations to target: *P* < 0.001 with Wilcoxon signed-rank test).

Median total DD was 12 g (IQR 12–16 g; range 6–16 g) and 12 g (IQR 8–16 g; range 4–18 g) on the first and second TDM measures, respectively. The variability in DD was 35% higher during the second TDM measures (*P* = 0.057; Figure S6). Taken together, the current TDM practice did not systematically alter average DD between first and second TDM but resulted in broader DD distribution, enhanced exposure precision and closer proximity of trough levels to 16 mg/L.

Among the 80 piperacillin treatment courses analysed with two TDM measurements, a dosage reduction was applied in 24/80 (30%), a dosage increase in 25/80 (31.25%) and no dosage change in 31/80 (38.75%). Among the 25 courses where the dosage was increased, an extension of the infusion duration (from 30 to 120 minutes) was also documented in four courses.

### Comparison between observed concentrations and model-based predictions

**Figure 3.**
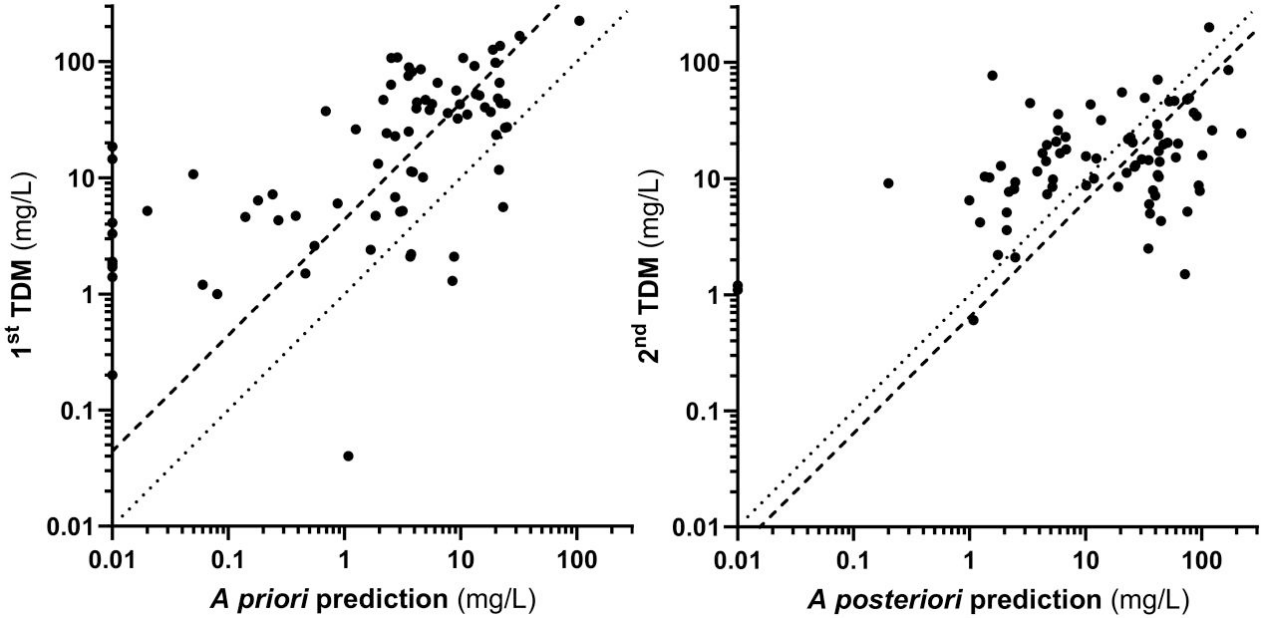
Concordance analysis between first TDM measurements and *a priori* predictions (left panel) and between second TDM measurements and *a posteriori* predictions (right panel). Dotted line: identity; dashed line: log-linear regression. The single point below the lower limit of quantification (LLOQ = 0.08 mg/L) was treated as LLOQ/2 (left panel).

Whilst piperacillin concentrations differed significantly between first TDM observations and *a priori* predictions from TUCUXI (median piperacillin trough concentration of 24.6 mg/L for the first TDM and of 3.7 mg/L for the *a priori* prediction; *P* < 0.001), the second TDM observations were more congruent with the *a posteriori* predictions from TUCUXI based on the first TDM measurements (median piperacillin trough concentration of 14.5 mg/L for the second TDM and of 24.9 mg/L for the *a posteriori* prediction based on the first TDM measurement; *P* = 0.01; Table S7 and Figure S7). The linear regression coefficient relating the first TDM with *a priori* predictions was 0.20 (95% CI 0.14–0.25), indicating a substantial discrepancy, whilst the MLE was −2.01 mg/L (range −7.52 to +3.29 mg/L) and the RMSLE 292%, further emphasizing the model's limitations (see Figure 3). Conversely, the integration of the first measured concentrations into the model notably improved the prediction performance: the linear regression coefficient between the second TDM measurements and *a posteriori* predictions (based on the first^t^ TDM measures) increased to 0.54 (95% CI 0.24–0.85), reflecting a positive adjustment in the model, and the MLE and RMSLE decreased to 0.24 mg/L (range −0.24 to +2.83) and 155%, respectively.

### Description and comparison of the six dosage adjustment strategies

**Figure 4.**
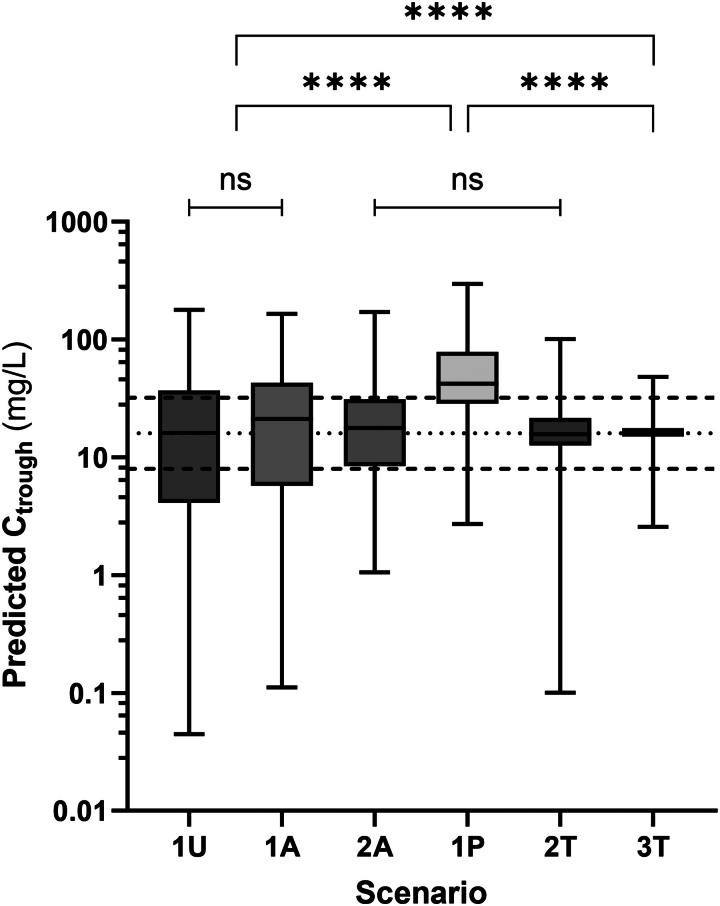
Predicted piperacillin trough concentrations with all six dosing strategies. Median concentration and proximity to 16 mg/L of scenario 1P are significantly higher in comparison with the five other dosing strategies (****P < 0.001). Significant differences in variance between the six strategies (****P < 0.001) with *post hoc* comparisons grouping strategies 1U and 1A together, strategies 2A, 1P and 2T together and leaving strategy 3T alone (see Text and Table S8).

Figure 4 illustrates how median trough concentrations (detailed in Table S8) fall within the target range of 8–32 mg/L, except for scenario 1P (*a priori* MIPD-based dosing strategy), which mostly overshoots the target.

**Figure 5.**
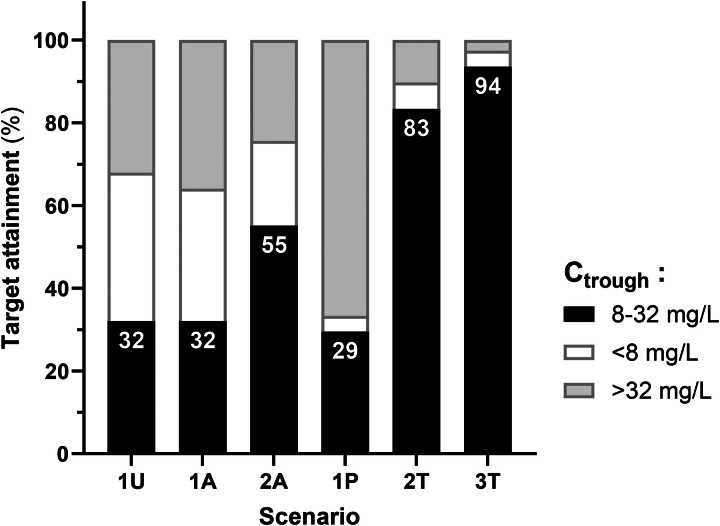
Probability of target attainment, defined as the percentage of total trough concentrations predicted in the 8–32 mg/L range, for the six dosing strategies. White and grey scares illustrate total trough concentrations predicted below and above this range.

Median DDs for the six dosage adjustment strategies are summarized in Table S9 and Figure S9. The variability in DDs correlated inversely with the variability of trough concentrations. The coverage of the whole dosage interval with at least 16 mg/L was the best for strategies 1P and 3T, while most strategies ensured minimum coverage of 8 mg/L and only strategy 1P ensured 32 mg/L in most patients (Table S10 and Figure S10). Assuming an optimal target range of 8–32 mg/L for trough concentrations (covering most usual bacteria identified in our centre), the PTA was 32%, 32%, 55%, 29%, 83% and 94%, respectively for the uniform dosage (1U), actual initial (chart-based) dosage (1A), actual dosage adjustment (2A), *a priori* MIPD-based dosage (1P), *a posteriori* MIPD-based dosage (2T) and best possible adjustment (3T) (*P* < 0.001, Cochran’s Q test; Figure 5). For comparison, we also performed the simulation of a uniform and more intensive dosage (4000 mg q6h, irrespective of renal function). The PTA (i.e. % trough concentrations predicted in the 8–32 mg/L range) for this scenario was 31%, with 20% of the results being <8 mg/L and 49% > 32 mg/L (compared with the 1U scenario of 4000 mg q8h, where PTA was 32% with 36% of the results <8 mg/L and 32% > 32 mg/L).

## Discussion

Our study included 78 patients, mainly in the ICU (59%), who had two piperacillin TDM measurements. Overall, observed trough total concentrations were within the target range (8–32 mg/L). Our data demonstrate how traditional TDM (consisting of measurement of concentrations and dosing adaptation according to pharmacologist's advice) enables better distribution of total DDs with enhanced precision and closer proximity to an ideal trough target (16 mg/L). However, exposure could be further improved with the systematic use of MIPD. Indeed, the comparison of observed concentrations with concentrations predicted by TUCUXI based on the popPK model of Chen *et al*.^19^ showed a clear improvement in prediction performance after integrating measured concentrations into the model. Initial overestimation of clearance associated with relying solely on patient covariates could be overcome and the model’s prediction performance improved after adding measured data. Among the six dosing strategies (identical dosage for all, chart-based dosage, actual empirical adjustment following first TDM, *a priori* MIPD-based dosage, *a posteriori* MIPD-based adjustment after first TDM and MIPD including both TDM measurements), all except *a priori* MIPD-based dosage were able to drive median trough concentrations within the target range of 8–32 mg/L. The dispersion of total DDs correlated inversely with the standard deviation of predicted trough concentrations, confirming that better precision means better distribution of doses between patients. In terms of antibiotic coverage, all feedback approaches improved PTA, which was poor in the absence of TDM (29%–32%). The similarity of PTA obtained with the same dosage for all patients (4000 mg q8h, irrespective of individual characteristics) and with chart-based dosing is explained by dosing recommendations being constant over a broad range of renal function according to the chart (Table S3). With a more intensive dosage (4000 mg q6h, irrespective of individual characteristics), the PTA remained quite similar to that of standard dosage (4000 mg q8h). TDM followed by dosing adjustment traditionally based on pharmacologist's advice significantly improved PTA (55%), while systematic MIPD-based dosing adjustment after TDM was expected to further increase PTA (up to 83%). The suboptimal performance of the *a priori* MIPD-based dosage scenario, particularly when compared with the chart-based scenario, may be attributed to the imperfect adequacy of the Chen *et al.*^19^ model for our study population. In particular, the dispersion of body weight and creatinine clearance values were larger in our patients (weight ranging from 49 to 140 kg and creatinine clearance from 9 to 230 mL/min). These extreme values might not be well-suited for the model developed by Chen *et al*.^19^ As shown in the supplementary data, the conditional weighted residuals (CWRES) values are not well centered around 0.

Our observations and simulations are in line with previous studies. Piperacillin TDM on its own has been demonstrated to lead to better target attainment than fixed dosage adjusted solely for renal function.^21^ Furthermore, several studies have established the potential benefit of MIPD over TDM alone. In a retrospective study of 179 patients admitted to ICU for sepsis and treated empirically with continuous piperacillin infusion, DDs were reduced by *a priori* MIPD prediction, and even further by incorporating TDM results.^22^ Whilst standard dosing alone led only 23% of patients within the therapeutic range, software-guided dosing increased this percentage to 40%, and incorporation of TDM to 65%. Our results tend to expand such findings to piperacillin administered via intermittent intravenous infusions. In another prospective study among critically ill patients, target attainment improved when combining TDM with a dosing software programme (ID-ODS), with 11 (85%) out of 13 patients with subtherapeutic piperacillin concentrations achieving target exposure.^15^ Finally, the DOLPHIN study was the first randomized clinical trial evaluating the clinical benefit of MIPD for various beta-lactams and ciprofloxacin among critically ill patients.^23^ It included 189 patients in the MIPD group and 199 patients in the standard care group. There was no difference in terms of length of ICU stay. Nonetheless, MIPD was able to improve the PTA (defined as fT > MIC for 100% of the dosing interval) from 55% to 70% after 7 days of treatment. Of note, our target range of 8–32 mg/L (based on local epidemiology) is lower than the targets typically proposed in the literature, which aim for a minimum of 100% fT > 4×MIC, particularly for ICU patients.^24^ Nevertheless, the demonstration that an MIPD-based approach allows for more precise achievement of target concentrations could equally apply to more aggressive targets.

Several limitations of our study need to be mentioned. First, it is based on retrospective data, with all potential biases associated with this design (data inaccuracies, selection of patients offered two TDM controls, absence of a control group, etc.). Second, it included a relatively small number of patients, with a risk of neglecting effects of limited size. Despite the limited inclusion period and sample size, the number of patients included was adequate to show a statistically significant benefit in achieving target concentrations with greater precision using an MIPD-based approach. This supports the use of MIPD in future, larger-scale prospective studies. Simulating TDM strategies inevitably relies on assumptions that cannot be formally proven, but which are largely supported by general PK knowledge. The model of Chen *et al*.^19^ might not be the best possible popPK model applying to our patients, as suggested by the poor performance of the *a priori* MIPD-based strategy (1P). Yet, its possible flaws did not impair the ability of *a posteriori* MIPD strategies to significantly improve the PTA, showing the robustness of a Bayesian approach even based on an imperfect prior. On the other hand, a bias in the extrapolations to trough, potentially resulting from model inaccuracy, would affect similarly the outcomes of all strategies compared. Recently, Schatz *et al*.^25^ have suggested to use a combination of available popPK models as prior rather than a single best model as prior for MIPD. Tazobactam was not measured in our study, which represents an additional limitation. Although tazobactam is administered with piperacillin at a fixed dose ratio of 1:8, piperacillin inhibits the renal excretion of tazobactam, and recent data have shown that this ratio is influenced by renal function.^26^ This raises concerns about the potential for tazobactam underdosing in patients with augmented renal clearance. Therefore, future dose adjustment strategies for piperacillin (whether based on TDM or MIPD approaches) should ideally take tazobactam concentrations into account. Only total concentrations, not free concentrations, were measured in our study. This represents an additional limitation, as free concentration is the pharmacologically active moiety. Nevertheless, total concentrations more closely reflect routine practice (free concentrations are not measured routinely). Furthermore, piperacillin’s protein binding exhibits considerable interindividual variability, especially in critically ill patients, and the correlation between albumin levels and free piperacillin concentrations is not well established.^27^ Therefore, we refrained from extrapolating an unbound fraction. A further limitation relates to the limited standardization of our current TDM practice, largely dependent on the expertise of the pharmacology consultant. Nevertheless, the actual PTA observed in our study is close to levels reported in the literature. Furthermore, many microbiological data were either missing or unavailable, particularly MIC values. As a result, we focused our study on a PK-based target approach rather than a PK/PD-based target approach. Finally, the most important study limitation is the lack of clinical outcomes, which precludes firm conclusions in terms of clinical efficacy, recovery, mortality, toxicity or length of hospital stay.

In conclusion, this study brings strong arguments for a better performance of TDM in terms of target attainment when combining it with MIPD. However, prospective studies remain needed to confirm the feasibility and benefits of MIPD, not only on target attainment but also on hard clinical outcomes.

## Supplementary Material

dkaf007_Supplementary_Data

## Supplementary data

Figures S1, S5–S7, S9, S10 and Tables S2, S3, S7–S10 are available as Supplementary data at *JAC* Online.
