# Supplementary material for: Individualization of piperacillin dosage based on therapeutic drug monitoring with or without model-informed precision dosing: a scenario analysis
Source: J Antimicrob Chemother. 2025 Jan 17;80(3):840–7. doi: 10.1093/jac/dkaf007 (PMC12086683; doi:10.1093/jac/dkaf007)
Supplement: dkaf007_Supplementary_Data [file dkaf007_Supplementary_Data.docx]

**SUPPLEMENTARY DATA: “INDIVIDUALIZATION OF PIPERACILLIN DOSAGE BASED ON THERAPEUTIC DRUG MONITORING WITH OR WITHOUT MODEL-INFORMED PRECISION DOSING: A SCENARIO ANALYSIS”**

David HAEFLIGER^1^, Lynn MINA^2^, Monia GUIDI^1,2^, Catia MARZOLINI^1,3,4^, Paul THOUEILLE^1^, Laura E. ROTHUIZEN^1^, Yann THOMA^5^, Laurent A. DECOSTERD^1^, Benoit GUERY^6^, François R. GIRARDIN^1^, Thierry BUCLIN^1^

Author affiliations:

1. Service of Clinical Pharmacology, Lausanne University Hospital and University of Lausanne, Lausanne, Switzerland.

2. Centre for Research and Innovation in Clinical Pharmaceutical Sciences, Lausanne University Hospital and University of Lausanne, Lausanne, Switzerland.

3. Division of Infectious Diseases and Hospital Epidemiology, University Hospital Basel, University of Basel, Basel, Switzerland.

4. Department of Molecular and Clinical Pharmacology, Institute of Translational Medicine, University of Liverpool, Liverpool, United Kingdom.

5. School of Engineering and Management Vaud, HES-SO University of Applied Sciences and Arts Western Switzerland, Yverdon-les-Bains, Switzerland.

6. Service of Infectious Diseases, Lausanne University Hospital and University of Lausanne, Lausanne, Switzerland.

**Supplement S1**

***Local epidemiology at the Lausanne University Hospital with MIC values of piperacillin for Enterobacteriaceae and Pseudomonas aeruginosa***

Figure S1: Distribution (%) of MIC values (mg/L) of piperacillin for strains of *Pseudomonas aeruginosa* and most Enterobacteriaceae (*Escherichia coli*, *Klebsiella pneumoniae*, *Klebsiella oxytoca*, *Klebsiella aerogenes*, *Hafnia alvei*, *Citrobacter koseri*, *Citrobacter freundii*, *Enterobacter cloacae*, *Morganella morganii*, *Proteus mirabilis*, *Proteus vulgaris,* *Serratia marcescens*) identified at the microbiology laboratory of Lausanne University Hospital through year 2021.

**Supplement S2**

***Identification of a suitable popPK model of piperacillin to implement in TUCUXI***

**Literature search:**

Literature search was performed in the PubMed, Web of Science and Embase databases, following a PRISMA procedure and a common search equation (MeSH terms): *piperacillin AND (population-pharmacokinetic* OR nonlinear-mixed-effect OR nonlinear-mixed-effects OR NONMEM OR MONOLIX) NOT (child*)*. Obtained duplicates were excluded. An initial selection was made based on the titles and abstracts of the publications identified, according to pre-determined inclusion criteria (population pharmacokinetic model, piperacillin, adult population, NONMEM or MONOLIX) and exclusion criteria (pediatric population, healthy volunteers, non-parametric analysis, continuous renal replacement therapy or continuous perfusion). For each population pharmacokinetic (popPK) model identified, a score was established based on the following criteria: type of modelling software and algorithm used; type of patients included and study performed; number of patients, samples and study compartments; covariates; description of the analytical and pharmacokinetic methods and results, and finally the internal and/or external validations performed. Table S2 summarizes the two models with the highest scores retained for external validation: Li *et al*. (2005)^[[1]](#footnote-1)^ and Chen *et al*. (2016)^[[2]](#footnote-2)^.

**Models in consideration:**

The popPK model of Chen *et al*. was built using data from a prospective study of patients known or suspected of nosocomial infection and hospitalized in a hospital affiliated to Soochow University (China). NONMEM software (version 7.1) was used with the FOCE algorithm to build the model. The popPK model of Li *et al*. was built on data from a randomized clinical trial including hospitalized patients with complicated intra-abdominal infection. NONMEM (version V, level 1.1, double precision) and the FOCEI algorithm were used to build the model. For these two popPK models, a one-compartment model best fitted with the data and the significant covariates were creatinine clearance (CrCl), calculated by the Cockcroft-Gault formula, and body weight for both of them. For the model of Chen *et al*., the population estimates of piperacillin PK parameters were 13.8 L/h for clearance (with 31.1 % inter-individual variability (IIV)) and 21.7 L for volume of distribution (with 38 % IIV). For the model of Li *et al*. these estimates were 13.8 L/h for clearance (with 34.6 % IIV) and 19.4 L for volume of distribution (with 40 % IIV).

Table S2: PopPK models retained for external validation

|  | Li *et al*. (2005) | Chen *et al*. (2016) |
| --- | --- | --- |
|  | Population pharmacokinetics and pharmacodynamics of piperacillin/tazobactam in patients with complicated intra-abdominal infection | Population pharmacokinetics and pharmacodynamics of piperacillin/tazobactam in patients with nosocomial infections |
| Modeling software used | NONMEM | NONMEM |
| Algorithms used | FOCEI ^(a)^ | FOCE ^(a)^ |
| Patient’s disease status | Target sick patients | Target sick patients |
| Study | Prospective | Prospective |
| Number of compartments | One-compartment model with linear elimination | One-compartment model with linear elimination |
| Number of patients | 56 | 50 |
| Number of samples | 184 | 590 |
| Pertinent tested covariates value ^(b)^ | 12 | 12 |
| Reporting of analytical methods (chemical) | Validated and published | Validated and published |
| Reporting of analytical methods (PK) | Detailed, validated, reproducible | Detailed, validated, reproducible |
| Results description | Model development table or detailed results | Model development table or detailed results |
| Internal validation | 2 internal validations | 3 internal validations |
| External validation | no | no |

a) FOCE stands for First Order Conditional Estimation and FOCEI stands for First Order Conditional Estimation with Interaction b) pertinent covariates (age, sex, body weight and CrCl) were *a priori* established according to available data on piperacillin PK.

**Population data:**

An external validation using data from patients who had piperacillin concentrations measured between January 2019 and November 2020 at our tertiary care centre was carried out to choose among these two models. Following data were collected: piperacillin dosage history (dose amounts, duration of infusions and intervals between doses), blood sample collection times, body weight, age, renal function estimated using the Cockcroft-Gault equation, albumin, and total protein. Pediatric patients, patients on hemodiafiltration and those on continuous infusion were excluded. Concentration values lacking information on the time of sampling, or the date and time of last piperacillin administration were also excluded. A total of 148 patients were screened and 76 patients (corresponding to 118 concentrations) could be included in the analysis. The table below describes their main characteristics.

| **Characteristics** | **Median (Min-Max) or percentage** |
| --- | --- |
| Female/Male (%) | 26/74 |
| Age (years) | 66 (19-88) |
| Weight (kg) | 74 (38-169) |
| CrCl (Cockcroft-Gault) (mL/min) | 60 (12-187) |

**External validation:**

An external validation of the two popPK models was carried out on NONMEM, fixing all the parameter values to their final estimations. Goodness-of-fit (GOF) diagnostic plots (observed vs individual predicted concentrations and conditional weighted residuals (CWRES) versus time after dose) were drawn. Mean errors (MLE) and root mean square errors (RMSLE) on log-transformed values were calculated. Resulting visual and numerical models’ comparison allowed for selection of the best model.

GOF plot for the model of Chen *et al*. (2016) with, on the left, the observed concentrations (DV) versus individual predicted concentrations (IPRED), and on the right, the conditional weighted residuals (CWRES) versus time after dose (TAD):

GOF plot for the model of Li *et al*. (2005) with, on the left, the observed concentrations (DV) versus individual predicted concentrations (IPRED), and on the right, the conditional weighted residuals (CWRES) versus time after dose (TAD):


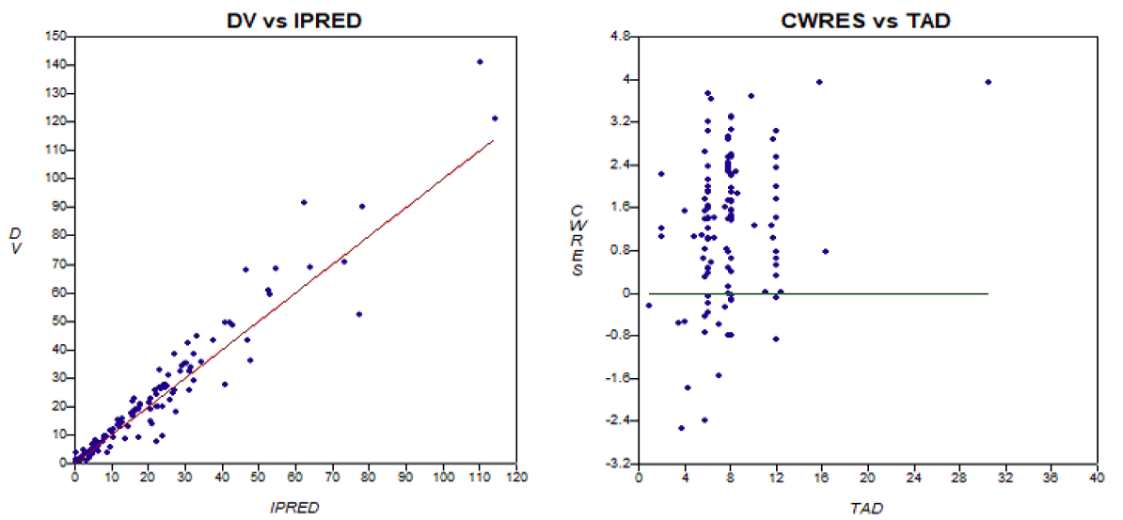


The popPK model of Chen *et al*. showed a non-significant MLE bias of +6% [0-11%] and a RMSLE accuracy of 33%, while the popPK model of Li *et al*. showed a significant MLE bias of -13% [-22, -2%] and a RMSLE accuracy of 88%. In view of these MLE and RMSLE values, the popPK model of Chen *et al*. was selected for validation in TUCUXI (even though the CWRES values were not well centered around 0 for both models).

**TUCUXI validation:**

The popPK model of Chen *et al*. was finally implemented in TUCUXI, whose computations were numerically validated using two methods.

The first method consisted in comparing the simulated concentration-time curves predicted by TUCUXI and NONMEM (i.e., comparison of TUCUXI's prediction with that of the gold standard used in popPK). For this step, python scripts (version 3.6) were run on a virtual machine with the Linux^®^ operating system (Ubuntu 18.04.4 LTS). One patient was randomly selected, and his dosing history and individual characteristics were used for the analysis. Based on these data and the TUCUXI and NONMEM scripts developed during this phase, 10’000 concentration profiles as a function of time were simulated over 80 hours, to compare the *a priori* predicted percentiles of the two software packages. The concordance of the predictions could be defined by visual inspection based on the superposition of the respective curves, visible on the various graphs mentioned above. Graphs of *a priori* percentiles obtained by NONMEM and TUCUXI were essentially superimposable (figure not shown).

The second method consisted in comparing the predictions calculated by NONMEM and TUCUXI of the last concentration of a few patients for whom several concentrations were available (n=16). The prediction of the last concentration was made based on all previous concentrations used to retrieve the *a posteriori* maximum likelihood individual PK parameter values in NONMEM and TUCUXI, which were then used to forecast the concentration at the time of the last measurement. These values were compared with NONMEM predictions calculated on the whole data set, computing the bias and accuracy (MLE and RMSLE) for NONMEM and TUCUXI. The direct comparison of the n-1 predictions between NONMEM and TUCUXI resulted in a MLE of 5% (-19;12) with a RMSLE of 38%.

**Supplement S3**

***Reference chart for the dosing of piperacillin***

The following recommendations have been adopted in the Lausanne University Hospital regarding the initial dosing of piperacillin, co-formulated with tazobactam and administered as intermittent intravenous infusions, as a function of the patient’s renal function. The adaptation of piperacillin dosage according to renal function has been devised based on a published consensus.^[[3]](#footnote-3)^ Piperacillin/tazobactam is indicated as empirical treatment in patients with sepsis, including septicemia, infections in immunocompromised or neutropenic patients, or serious infections of the respiratory tract, kidney, or urinary tract, or severe intra-abdominal or soft tissues infections.

Table S3: Institutional dosage recommendations for intravenous piperacillin.

| IV piperacillin (30 minutes infusion) | eGFR > 90 mL/min/1.73m^2^ | eGFR 89-60 mL/min/1.73m^2^ | eGFR 59-30 mL/min/1.73m^2^ | eGFR 29-15 mL/min/1.73m^2^ | GFR < 15 mL/min/1.73m^2^ |
| --- | --- | --- | --- | --- | --- |
| standard dosage | 4000 mg q8h | 4000 mg q8h | 4000 mg q12h | 2000 mg q8h | 2000 mg q12h |
| *Pseudomonas aeruginosa* infection (suspected/documented) | 4000 mg q6h | 4000 mg q6h | 4000 mg q8h | 4000 mg q12h | 2000 mg q8h |

**SUPPLEMENT S4**

***NONMEM code for the population pharmacokinetic model of piperacillin***

The individual *a posteriori* maximum likelihood parameter values of each study patient were calculated with NONMEM from their TDM measurements, starting from the *a priori* parameter distributions given in the model of Chen *et al*. The model then used these individual parameters to predict concentration profiles and trough values expected under the different dosing strategies compared in our study (the instruction MAXEVAL=0 enables to use NONMEM only for applying the model to the study patients, without re-estimating the PK parameters). The NONMEM code for the model of Chen *et al*. is listed below; it follows the usual language standards of NM-TRAN:

$PROBLEM PIPERACILLIN POPULATION PK MODEL OF CHEN 2016

$INPUT ID EVID TIME CMT AMT TINF RATE ADDL II SS DV MALE WT CLCR

$DATA Piperacilline_data.csv IGNORE=#

$SUBROUTINES ADVAN1 TRANS2

$PK

FCLCR = CLCR/68.7

IF (CLCR.LT.0) FCLCR = 64.96/68.7

FWT = WT/61.1

IF (WT.LT.0) FWT = 75.25/61.1

TVCL = THETA(1) + THETA(2)**(FCLCR)

CL = TVCL * EXP(ETA(1))

TVV = THETA(3) + THETA(4)**(FWT)

V = TVV * EXP(ETA(2))

S1 = V/1000

$ERROR

IPRED = F

DEL=0

IF (IPRED.EQ.0) DEL=1

W = IPRED + DEL

Y = IPRED + W*EPS(1)

IRES = DV-IPRED

IWRES = IRES/W

$THETA

(9.14) ; CL

(4.60) ; Impact of CLCR on CL

(12.2) ; V

(9.49) ; Impact of WT on V

$OMEGA

(0.0967) ; IIV CL

(0.1444) ; IIV V1

$SIGMA

0.0087 ; Proportional error PK

$EST METHOD=1 INTER MAXEVAL=0 NOABORT SIG=5 PRINT=1 POSTHOC

$TABLE ID EVID TIME DV PRED IPRED RES IRES WRES IWRES CWRES ONEHEADER NOPRINT FILE=sdtab01

$TABLE ID CL V MALE WT CLCR ONEHEADER NOPRINT FILE=patab01

**Supplement S5**

***Study flow chart***

The following flow chart shows all identified patients through the year 2021 with measurement of piperacillin concentrations. After applying the inclusion/exclusion criteria, 78 patients could be retained (with two patients included several weeks/months apart). The final analysis thus covered 80 courses of piperacillin with two TDM measurements.

Exclusion:

- 5 patients with missing data

- 2 patients with extreme values of creatinine clearance not fitting with the popPK model of Chen *et al*.

261 adult patients (> 18 years old) with measurements of piperacillin concentrations throughout the year 2021 (total of 519 TDM values)

Exclusion:

- 112 patients with only one concentration measurement

- 36 patients undergoing CRRT (continuous or intermittent dialysis)

- 20 patients undergoing CRRT with only one concentration measurement

- 8 patients receiving continuous intravenous administration

85 patients with 2 measurements of piperacillin concentration

78 patients with 2 measurements of piperacillin concentrations (2 patients included at several weeks/months apart) = 80 courses of piperacillin with 2 TDM measurements

Identificationc

Eligibility

Analysis

**SUPPLEMENT S6**

***Comparison of daily doses on first and second TDM controls***

Median total daily dose (DD) was 12 g (IQR 12-16 g; range 6-16 g) and 12 g (IQR 8-16 g; range 4-18 g) on the 1^st^ and 2^nd^ TDM measures respectively. The difference was not statistically significant (p=0.937 using two-sample Wilcoxon rank-sum test). Nevertheless, the variability in dosage regimens observed during the 2^nd^ TDM measure tended to be larger than on the 1^st^ TDM (variance ratio F=0.65, p=0.057).

Figure S6: Distribution of total daily doses on the 1^st^ and 2^nd^ TDM.

**SUPPLEMENT S7**

***Comparison of observations and predictions***

Comparing observed piperacillin concentrations (1^st^ and 2^nd^ TDM measures) and model-based predicted piperacillin concentrations, Table S7 and Figure S7 summarize the results. Using paired Wilcoxon signed-rank test, the difference of median piperacillin concentrations was statistically significant between first observations and *a priori* predictions (p<0.001, ***). The difference of median piperacillin concentrations remained statistically significant between second observations and *a posteriori* predictions based on the 1^st^ TDM measures (p=0.01, *), but the effect size was smaller.

|  | **Median piperacillin concentration** (mg/L) | **Interquartile range** (mg/L) | **p value** |
| --- | --- | --- | --- |
| 1^st^ TDM | 24.6 | 4.7 - 48.2 | <0.001 |
| *A priori* prediction | 3.7 | 0.9 - 11.4 |  |
| 2^nd^ TDM | 14.5 | 7.9 - 24.5 | 0.01 |
| *A posteriori* prediction | 24.9 | 4.7 – 46.8 |  |


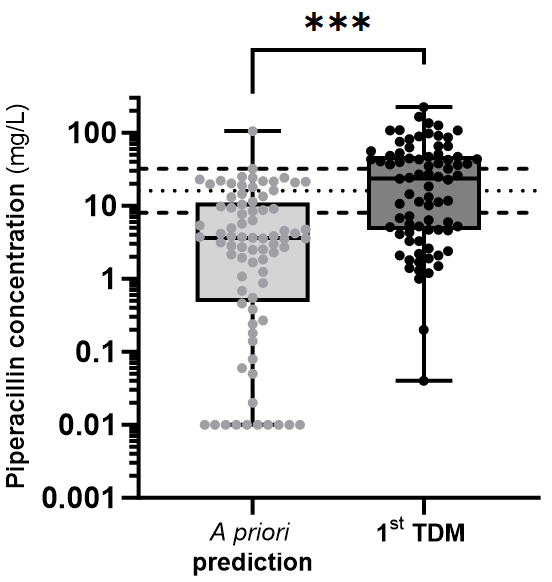


**SUPPLEMENT S8**

***Comparison of trough concentrations between the dosing strategies***

Comparing the six dosage adjustment strategies, namely **1U** for uniform dosage, **1A** for actual initial dosage (chart-based), **2A** for actual dosage adjustment (following 1^st^ TDM), **1P** for *a priori* MIPD-based dosage, **2T** for *a posteriori* MIPD-based dosage, and **3T** for the best possible adjustment (two MIPD cycles), Table S8 summarizes the median values for the predicted trough piperacillin concentrations obtained under each dosage adjustment scenario at steady-state.

| **Dosage adjustment scenario** | **Median piperacillin trough concentration** (mg/L) | **Interquartile range** (mg/L) | **95% Confidence interval** (mg/L) |
| --- | --- | --- | --- |
| 1U | 16.1 | 4.2 – 36.4 | 10.1 – 25.3 |
| 1A | 21.2 | 6.0 – 42.8 | 10.6 – 30.4 |
| 2A | 17.7 | 8.4 – 30.7 | 13.9 – 21.0 |
| 1P | 42.2 | 28.5 – 77.6 | 35.1 – 49.7 |
| 2T | 15.7 | 12.6 – 21.4 | 14.4 – 17.9 |
| 3T | 16.4 | 15.1 – 17.6 | 15.8 – 16.9 |

Concentrations were compared by means of an analysis of variance (ANOVA) using log-transformed concentration values and showing a significant difference among the various scenario groups (5 degrees of freedom, F = 20.3, p<0.001). Post-hoc analysis with Tukey test identified only scenario 1P (*a priori* MIPD-based dosage) out from all other scenarios, which were not statistically different from each other in terms of average concentrations. Variances were greatest for scenarios **1U** (uniform dosage) and **1A** (actual initial dosage) and smallest for scenario **3T** (best possible adjustment), while variances of scenarios **2A** (actual dosage adjustment), **1P** (*a priori* MIPD-based dosage) and **2T** (*a posteriori* MIPD-based dosage) were quite similar and intermediate between these two extremes. Indeed, Levene's test for equality of variance revealed highly significant differences in variance between the six scenarios (p<0.001). Group-by-group post-hoc comparisons using F-tests and a Bonferroni correction for multiplicity grouped together scenarios **1U** (uniform dosage) and **1A** (actual initial dosage), as well as scenarios **2A** (actual dosage adjustment), **1P** (*a priori* MIPD-based dosage) and **2T** (*a posteriori* MIPD-based dosage), into groups of non-different variance; and scenario **3T** (best possible adjustment) alone defined a third group. Between these groupings, the differences in variances were significant (p<0.001). In view of proximity of predicted piperacillin trough concentrations to ideal target of 16 mg/L, scenario **1P** showed greater distance to this target than all five other scenarios, which didn’t differ from each other (ANOVA with 5 degrees of freedom, F = 10.2, p<0.001 and post-hoc analysis with Tukey test).

**SUPPLEMENT S9**

***Comparison of daily doses between the dosing strategies***

Table S9 summarizes the distribution of daily doses respectively recommended by the six dosage adjustment strategies, namely the uniform dosage (**1U**), the actual initial dosage (**1A**), the actual dosage adjustment (**2A**), the *a priori* MIPD-based dosage (**1P**), the *a posteriori* MIPD-based dosage (**2T**) and the best possible adjustment (**3T**).

| **Dosage adjustment scenario** | **Total daily**  **dose** (g) | **Interquartile**  **range** (g) | **95% Confidence Interval** (g) |
| --- | --- | --- | --- |
| 1U | 12 | 12-12 | 12-12 |
| 1A | 12 | 12-16 | 12-12 |
| 2A | 12 | 8-16 | 12-16 |
| 1P | 16 | 12-18 | 12-18 |
| 2T | 9 | 6-16 | 8-12 |
| 3T | 8 | 6-12 | 8-9 |

As illustrated in Figure S9 below, dosage schemes based on MIPD provide a lower total daily dose along with a greater variability. Indeed, average daily doses and their variances differed significantly between the six different scenarios by means of ANOVA (p<0.001) and Levene’s test (p<0.001), respectively. There was a significant inverse correlation between the standard deviation (SD) of the daily doses and the SD of the predicted trough concentrations (log-transformed values), indicating that, as expected, the variability of concentrations evolved inversely to the variability of daily doses.

**SUPPLEMENT S10**

***Comparison of concentration coverage between the dosing strategies***

Table S10 show the percentage (mean % and SD) of the dosing interval where concentrations exceeded 8, 16 or 32 mg/L, respectively for the six dosing strategies. The box-plots in Figures S10 represent the corresponding medians, interquartile ranges and spans. Comparison of means and variances showed that coverage differed significantly between the six dosing strategies (ANOVA and Levene’s test both with p<0.001), with better coverage obtained with TDM dosage adjustments, and TDM with MIPD dosage adjustments.

| **Dosage adjustment scenario** | **1U** | **1A** | **2A** | **1P** | **2T** | **3T** |
| --- | --- | --- | --- | --- | --- | --- |
| % of dosing interval > 8 mg/L | 88% (20) | 92% (15) | 96% (10) | 98% (12) | 98% (13) | 99% (5) |
| % of dosing interval > 16 mg/L | 79% (27) | 82% (24) | 88% (19) | 97% (14) | 90% (19) | 92% (20) |
| % of dosing interval > 32 mg/L | 64% (33) | 67% (33) | 65% (30) | 81% (36) | 53% (31) | 50% (26) |

1. Li C, Kuti JL, Nightingale CH, Mansfield DL *et al*. Population pharmacokinetics and pharmacodynamics of piperacillin/tazobactam in patients with complicated intra-abdominal infection*. J Antimicrob Chemother*. 2005; **56**: 388-95. doi: 10.1093/jac/dki243. [↑](#footnote-ref-1)
2. Chen R, Qian Q, Sun MR *et al*. Population Pharmacokinetics and pharmacodynamics of piperacillin/tazobactam in patients with nosocomial infections. *Eur J Drug Metab Pharmacokinet*. 2016; **41**: 363-72. doi: 10.1007/s13318-015-0276-3. [↑](#footnote-ref-2)
3. Willi-Robatel C, Senn L, Buclin T *et al*. Agents anti-infectieux et fonction rénale: vers des posologies sur mesure ? [Antimicrobial agents and renal elimination: towards individual dosage adjustment?]. *Rev Med Suisse*. 2012; **8**: 894-900. doi : 10.53738/REVMED.2012.8.338.0894. [↑](#footnote-ref-3)
